# Positive effect of intravenous iron-oxide administration on left ventricular remodelling in patients with acute ST-elevation myocardial infarction - a cardiovascular magnetic resonance (CMR) study

**DOI:** 10.1186/1532-429X-16-S1-O24

**Published:** 2014-01-16

**Authors:** Anca Florian, Anna Ludwig, Sabine Rösch, Handan Yildiz, Udo Sechtem, Ali Yilmaz

**Affiliations:** 1 Cardiology and Angiology, University Hospital Münster, Münster, Germany; 2 Cardiology, Robert-Bosch-Krankenhaus, Stuttgart, Germany

## Background

This study investigated the safety profile and potential "therapeutic" effect of intravenous ultrasmall superparamagnetic iron-oxide (USPIO)-based iron administration regarding infarct healing in patients with ST-elevation myocardial infarction (STEMI).

## Methods

Seventeen study patients (IRON, 54 ± 9 yrs, 88% male) and 22 matched controls (CONTROL, 57 ± 9 yrs, 77% male) both with primary reperfused STEMI underwent multi-parametric CMR studies in the first week and three months after acute MI. Only IRON patients received a single intravenous bolus of 510 mg elemental iron as ferumoxytol (FerahemeTM) within four days following acute MI.

## Results

Three months after study inclusion, all patients were alive and there were no adverse cardiac events. Significant improvement in left ventricular (LV) ejection fraction (IRON: 53 ± 10% to 59 ± 9%, p = 0.002; CONTROL: 54 ± 6% to 57 ± 10%, p = 0.005) as well as shrinkage of infarct size were seen in both groups at follow-up. There was a more pronounced decrease in infarct size in the IRON group (IRON: -10.3 ± 5.4% vs. CONTROL: -7.0 ± 8.4%, p = 0.050) in addition to a significant decrease in both endocardial extent and prevalence of transmural infarctions in IRON but not in CONTROL patients. A significant decrease in LV end systolic volume was only seen in the IRON group (71 ± 25 mL to 59 ± 25 mL, p = 0.002).

## Conclusions

Intravenous iron administration in acute STEMI patients is associated with both improved infarct healing and beneficial global left ventricular remodelling (compared to matched controls). These findings together with the good safety profile make USPIO-based iron administration a promising future candidate as a "diagnostic" and "therapeutic" adjunctive solution in acute MI management.

## Funding

This work was financially supported by a grant from the German Federal Ministry of Education and Research (BMBF; grant-ID 01EZ0818 to A.Y. and U.S.).

